# Comparative Effectiveness of Conservative Therapies for Plantar Fasciitis: A Retrospective Observational Study

**DOI:** 10.3390/sports13090306

**Published:** 2025-09-05

**Authors:** Ana María Rayo-Pérez, José María Juárez-Jiménez, Mercedes Ortiz-Romero, Luis María Gordillo-Fernández, Raquel García-De-La-Peña

**Affiliations:** Department of Podiatry, University of Seville, 41009 Seville, Spain; anarayo43@gmail.com (A.M.R.-P.);

**Keywords:** plantar fasciitis, heel pain, physical therapy, shockwave therapy, conservative treatment

## Abstract

**Background:** Plantar fascitis is a common cause of heel pain in adults. Although various conservative treatments have been studied, comparative real-world effectiveness remains underreported. **Objective:** To retrospectively evaluate and compare the clinical effectiveness of percutaneous neuromodulation, extracorporeal shockwave therapy (ESWT), and custom foot orthoses in patients with plantar fasciitis. **Methods:** A retrospective observational study was conducted from January 2020 to December 2023 at a podiatric clinic. Inclusion and exclusion criteria were applied to medical records, and 120 patients were divided into three groups according to treatment, with 7 patients excluded due to non-compliance with follow-up. Pain intensity (VAS) and functional improvement (FFI) were assessed at baseline, 1 month, and 6 months post-treatment. Statistical analysis included paired t-tests, ANOVA, effect size (Cohen’s d), and 95% confidence intervals. The STROBE checklist was followed. **Results:** All three interventions showed significant improvement in pain and function at 6 months (*p* < 0.05). Neuromodulation achieved the highest pain reduction (VAS mean difference −6.2, d = 1.02), followed by ESWT (d = 0.78) and orthoses (d = 0.65). Functional scores improved similarly across all groups, with no significant difference at 6 months (*p* = 0.12). **Conclusions:** Percutaneous neuromodulation demonstrated greater clinical effectiveness in pain reduction compared to ESWT and orthoses, although functional outcomes were similar. Further prospective studies are needed to confirm these findings.

## 1. Introduction

Plantar fasciitis is a leading cause of heel pain in adults and a prevalent musculoskeletal condition in clinical settings, particularly among physically active individuals and workers who spend long hours standing. Epidemiological data estimate that approximately 10–20% of the general population will experience this condition at some point in their lives [1]. Clinically, it manifests as sharp, localized pain in the inferomedial region of the heel, typically exacerbated during the first steps in the morning or after periods of rest. Contrary to its traditional inflammatory conceptualization, histopathological studies suggest a chronic degenerative process of the plantar fascia, which has led some authors to adopt the term “plantar fasciosis” [1,2].

Anatomically, the plantar fascia is a fibrous aponeurosis extending from the medial calcaneal tubercle to the metatarsal heads. It plays a critical role in maintaining the integrity of the medial longitudinal arch and in absorbing and redistributing mechanical loads during gait [3]. Biomechanical alterations—such as rearfoot overpronation, gastrocnemius-soleus tightness, or intrinsic foot muscle weakness—can predispose the fascia to repetitive microtrauma and collagen degeneration [3,4,5].

Several risk factors have been identified, including obesity, prolonged standing, high-impact activities, inadequate footwear, and systemic inflammatory diseases such as spondyloarthropathies [4,6]. Conservative treatment remains the first-line approach, comprising rest, footwear modification, analgesia, physiotherapy, and orthotic devices [7,8]. Customized insoles and medial wedges have shown benefit in improving load distribution, correcting biomechanical dysfunction, and reducing pain [5].

Physical therapy strategies, including eccentric strengthening, myofascial release, manual therapy, and physical agents such as ultrasound or laser, are frequently used. A recent meta-analysis supports the effectiveness of dry needling combined with stretching in reducing symptoms and improving ultrasonographic features of the fascia [8]. Moreover, neuromodulatory approaches such as extracorporeal shockwave therapy (ESWT) have gained recognition for their capacity to reduce pain through mechanical stimulation and modulation of nociceptive pathways [9].

Alternative therapies such as platelet-rich plasma (PRP) or high-intensity laser therapy (HILT) have also shown promising results [10,11,12], although heterogeneity in protocols limits generalization. When conservative measures fail—reported in up to 10% of cases—minimally invasive or surgical interventions may be required [6,13].

In recent years, neuromodulation has emerged as a non-invasive therapeutic modality that aims to restore function by altering afferent and efferent nerve activity. Preliminary studies suggest that neuromodulation may improve outcomes in chronic musculoskeletal pain, including plantar fasciitis, although evidence remains limited and heterogeneous [9].

Despite the breadth of available treatments, there is a lack of comparative studies evaluating the real-world effectiveness of neuromodulation, ESWT, and orthotic interventions under uniform clinical conditions. Furthermore, the placebo effect, reported to account for up to 30% of symptom improvement [14], underscores the need to discern between statistical significance and clinically meaningful benefit.

The primary objective of this study is to compare the clinical effectiveness of neuromodulation, extracorporeal shockwave therapy, and customized orthoses in patients with plantar fasciitis, based on pain reduction and functional improvement. Secondary objectives include assessing the clinical relevance of outcomes using effect size estimations and determining whether any of the interventions provide superior results.

## 2. Materials and Methods

### 2.1. Study Design and Experimental Approach

This retrospective, observational, and comparative study analyzed clinical records from adult patients diagnosed with plantar fasciopathy—a term encompassing conditions historically referred to as *plantar fasciitis*, *fasciosis*, or *plantar heel pain syndrome*. For clarity, the term *plantar fasciopathy* will be used throughout, reflecting current clinical guideline recommendations [1,2]. The study aimed to evaluate and compare the short-term (1 month) and long-term (6 months) effectiveness of three conservative physical therapy modalities—percutaneous neuromodulation, extracorporeal shockwave therapy (ESWT), and custom-made foot orthoses—when applied prior to or in conjunction with ultrasound-guided collagen injections.

The study followed the STROBE (Strengthening the Reporting of Observational Studies in Epidemiology) recommendations for observational research.

### 2.2. Population and Eligibility Criteria

Inclusion and exclusion criteria are presented in Table 1 for clarity.

Patients were treated between January 2020 and December 2023 at a specialized outpatient podiatry clinic in Seville, Spain, affiliated with the Clínica Rayo.

### 2.3. Sample Size Calculation

Based on prior studies, a minimum clinically important difference of 1.2 points in VAS scores (standard deviation: 1.8) was assumed. To detect this difference with 80% power, α = 0.05, and a 95% confidence level, a sample size of 50 patients per group (total N = 150) was required. However, during the recruitment period, only 120 patients were enrolled. After applying the exclusion criteria and accounting for loss to follow-up, the final sample comprised 113 participants, who were evenly distributed across the three groups.

### 2.4. Description of Interventions

Group 1: Percutaneous Neuromodulation + Collagen Injection. Neuromodulation device: PhysioInvasiva**^®^** PN-2000 (Prim Fisioterapia, Madrid, Spain), applied on days 0, 7, 14; collagen injection (TendoVis**^®^**, IBSA, Lugano, Switzerland) under ultrasound guidance on day 21; further neuromodulation sessions on days 7, 14, and 21 post-injection.

Group 2: ESWT + Collagen Injection. ESWT device: Swiss DolorClast**^®^** Smart 20 (EMS, Nyon, Switzerland), 2.0–3.0 bar, 2000 impulses/session, on days 0, 7, 14; collagen injection on day 21; further ESWT sessions on days 7, 14, and 21 post-injection.

Group 3: Custom-Made Foot Orthoses + Collagen Injection. Collagen injection (MD, Oyasama, Madrid, Spain) on day 0; orthoses fitted on day 14 (polypropylene shell, EVA cover, 4° medial rearfoot post, MD, Oyasama, Madrid, Spain); second collagen injection on day 21.

### 2.5. Definition of Therapeutic Failure

Patients with persistent symptoms after 6 months were considered non-responders. These cases were offered ultrasound-guided percutaneous fasciotomy with platelet-rich plasma (PRP) application, as part of a second-line intervention pathway.

### 2.6. Study Variables

The primary independent variable in this study was the type of conservative treatment administered, categorized into three groups: percutaneous neuromodulation, extracorporeal shock wave therapy (ESWT), or custom-made foot orthoses. The primary dependent variables were pain intensity, measured using the Visual Analog Scale (VAS, range 0–10), and functional limitation, assessed through the Foot Function Index (FFI). Both outcomes were recorded at baseline, one month, and six months following treatment.

Secondary outcomes included the need for additional therapeutic interventions, patient satisfaction—evaluated using a structured questionnaire at the end of the follow-up period—and the presence of any adverse effects or complications related to the treatments received.

Potential confounding variables considered in the analysis were patient age and sex, body mass index (BMI), duration of symptoms prior to treatment initiation, and the laterality of the condition (unilateral or bilateral involvement).

### 2.7. Data Collection Procedure

Data were extracted from electronic medical records using a standardized data abstraction form by two independent reviewers. Discrepancies were resolved by consensus. All outcome variables and time points were verified, and missing data were handled through complete case analysis (no imputation).

### 2.8. Statistical Analysis

Descriptive statistics were employed to summarize the demographic and clinical characteristics of the study population. Continuous variables were presented as means with standard deviations (SD) or as medians with interquartile ranges (IQR), depending on data distribution, while categorical variables were expressed as frequencies and percentages. Between-group comparisons were conducted using one-way analysis of variance (ANOVA) or the Kruskal–Wallis test for continuous variables, and the chi-square or Fisher’s exact test for categorical variables, as appropriate. Longitudinal outcome data were analyzed using repeated-measures ANOVA to assess within- and between-group effects over time.

Effect sizes for the primary outcomes were calculated using Cohen’s d, along with corresponding 95% confidence intervals to estimate the magnitude and precision of the observed effects. To control for potential confounding factors, multivariate regression models were applied. A two-sided *p*-value of less than 0.05 was considered statistically significant. All analyses were performed using the latest version of IBM SPSS Statistics version 27.

### 2.9. Ethical Considerations

This retrospective study complied with the ethical principles of the Declaration of Helsinki. As no additional interventions were performed and all data were anonymized, the study was exempt from formal ethical board approval under local regulations.

During initial consultations, all patients signed an informed consent form that included authorization for the use of anonymized data, images, and videos for research and academic purposes. At no point were personal identifiers accessed or used during the data analysis process.

## 3. Results

### 3.1. Descriptive Statistics

Based on the a priori sample size calculation, a total of 150 patients (50 per group) was required to achieve 80% power (β = 0.20; α = 0.05) to detect a minimum clinically important difference of 1.2 points in VAS scores (SD = 1.8). During the recruitment period, 120 patients were initially enrolled. After applying the exclusion criteria and accounting for loss to follow-up, the final sample comprised 113 patients diagnosed with plantar fasciopathy, who were distributed across the three treatment groups: neuromodulation (n = 35), extracorporeal shock wave therapy (ESWT; n = 40), and custom orthotic supports (n = 38).

Since the achieved sample size was lower than initially planned (approximately 37–40 patients per group instead of 50), the statistical power for detecting the interaction effect in the repeated-measures ANOVA was reduced. A post hoc power analysis was therefore performed for the interaction term, based on the observed effect size and variance structure of the data. The observed power was estimated at 0.68, indicating that the study was slightly underpowered compared to the originally targeted 0.80. This limitation should be considered when interpreting the results, particularly for the interaction effects across treatment groups over time.

The mean age of participants was 47 years (range 29–67), with 58% of the sample being male and 42% being female. The average body mass index (BMI) was 28.5 (range 22.1–32.5), indicating a predominance of overweight individuals. The mean symptom duration was 14 months (range 5–25 months).

Baseline characteristics showed no significant differences between groups in terms of age (p = 0.62, F(2,110) = 0.48, partial η^2^ = 0.009), BMI (p = 0.85, F(2,110) = 0.16, partial η^2^ = 0.003), symptom duration (p = 0.91, F(2,110) = 0.10, partial η^2^ = 0.002), or sex distribution (p = 0.89, χ^2^(2) = 0.22, φ = 0.044), confirming group homogeneity at baseline (Table 2).

### 3.2. Clinical Outcomes Assessment

Pain intensity (VAS) and foot function (FFI) were assessed at baseline, 1 month, and 6 months post-treatment (Table 3 and Table 4).

All groups showed significant reductions in pain over time (*p* < 0.001), with large effect sizes observed (partial η^2^ ranged from 0.49 to 0.54). Initial mean VAS scores were similar across groups (~7.8). At six months, the neuromodulation group reported the greatest pain reduction (1.5 ± 1.4, 95% CI 1.0–2.0), followed by ESWT (1.8 ± 1.6, 95% CI 1.3–2.3) and orthotic supports (1.9 ± 1.5, 95% CI 1.4–2.4). Functional improvements measured by FFI were also significant in all groups (*p* < 0.001, partial η^2^ from 0.50 to 0.56). Baseline FFI scores ranged from 75 to 76, decreasing to 30 (95% CI 26–34) in the neuromodulation group, 32 (95% CI 29–35) in the ESWT group, and 33 (95% CI 30–36) in the orthotic group at six months.

Patient satisfaction at six months was highest in the neuromodulation group (4.2 ± 0.9, 95% CI 3.8–4.6), followed by ESWT (4.0 ± 0.8, 95% CI 3.7–4.3) and orthotic supports (3.9 ± 0.7, 95% CI 3.6–4.2), though differences were not statistically significant (*p* = 0.17, partial η^2^ = 0.03).

### 3.3. Normality Tests

The Shapiro–Wilk test confirmed normal distribution of VAS and FFI scores across all time points (*p* > 0.05), validating the use of parametric statistical methods.

### 3.4. Inferential Analysis

Between-group comparisons at six months via one-way ANOVA revealed significant differences for pain (VAS) (F(2,110) = 4.10, *p* = 0.02, partial η^2^ = 0.07), but not for function (FFI) (F(2,110) = 2.10, *p* = 0.12, partial η^2^ = 0.04) or patient satisfaction (F(2,110) = 1.80, *p* = 0.17, partial η^2^ = 0.03).

Post hoc Tukey tests indicated that neuromodulation produced significantly greater pain reduction than both ESWT (*p* = 0.03) and orthotic supports (*p* = 0.01) (Table 5).

### 3.5. Correlations Between Variables

A weak but statistically significant negative correlation was found between BMI and VAS improvement (r = –0.21, *p* = 0.03), indicating that higher BMI was associated with smaller reductions in pain. Symptom duration showed a moderate positive correlation with residual pain (r = 0.35, *p* < 0.001), suggesting lower treatment efficacy in patients with longer symptom histories.

### 3.6. Effect Size

Cohen’s d analysis demonstrated small-to-moderate effect sizes favoring neuromodulation over the other treatments:

Neuromodulation vs. ESWT: *d* = 0.45
Neuromodulation vs. Orthotic Supports: *d* = 0.52

These effect sizes indicate a clinically meaningful advantage of neuromodulation in pain reduction and functional improvement.

## 4. Discussion

Plantar fasciopathy is one of the most common causes of heel pain, particularly among active adults and overweight individuals. This study compared the effectiveness of three widely used non-invasive treatments—neuromodulation, extracorporeal shock wave therapy (ESWT), and custom plantar orthotics—assessing their impact on pain, foot function, and patient satisfaction over a six-month period. The findings contribute valuable evidence to the non-surgical management of this prevalent condition.

Consistent with previous research, including the meta-analysis by Charles et al. [15] and clinical practice guidelines such as those from Koc et al. [16] and Morrissey et al. (2021) [17], which highlight ESWT’s efficacy in tendinopathies and plantar fasciopathy, all treatment groups in our study showed significant clinical improvements in pain and function over time (*p* < 0.001, partial η^2^ ranging from 0.49 to 0.56). Nevertheless, neuromodulation produced a statistically significant greater reduction in pain at six months (VAS: 1.5 ± 1.4, 95% CI 1.0–2.0) compared to ESWT (1.8 ± 1.6, 95% CI 1.3–2.3) and plantar orthotics (1.9 ± 1.5, 95% CI 1.4–2.4) (*p* = 0.02, partial η^2^ = 0.07). While the effect size indicates a small to moderate clinical advantage, these results highlight the potential relative benefit of neuromodulation in managing plantar fasciopathy pain.

Supporting the effectiveness of ESWT, prior studies by Melese et al. [18] and Nazim et al. [19] reported significant improvements in pain and function in patients with chronic plantar fasciopathy. However, differences in treatment protocols, energy dosages, and devices likely contribute to heterogeneity among study outcomes, as emphasized by de la Corte-Rodríguez et al. [20], which limits direct comparability and underscores the need for standardized treatment guidelines.

Regarding custom plantar orthotics, although significant improvements in pain and function were observed, the magnitude was comparatively less pronounced. This aligns with Buchanan et al. [21], who note that orthoses provide symptomatic relief, but their effectiveness is influenced by individual factors such as foot morphology, symptom severity, and patient adherence.

Patient satisfaction was highest in the neuromodulation group (4.2 ± 0.9, 95% CI 3.8–4.6), although differences with ESWT (4.0 ± 0.8) and orthotics (3.9 ± 0.7) were not statistically significant (*p* = 0.17, partial η^2^ = 0.03). This trend may reflect patients’ perception of more rapid or sustained symptom relief with neuromodulation, a phenomenon documented in other musculoskeletal conditions treated with similar technologies.

Correlation analyses revealed clinically relevant associations: a negative correlation between BMI and pain improvement (r = −0.21; *p* = 0.03) suggests that patients with higher BMI experienced less pain reduction, consistent with Noriega et al. [22], who identified overweight and obesity as risk factors for persistent plantar pain. Additionally, symptom duration positively correlated with residual pain at six months (r = 0.35; *p* < 0.001), reinforcing the importance of early intervention, as also highlighted by Boonchum et al. [23] in studies on early treatments such as dynamic taping.

Other conservative treatments, including plantar fascia and calf muscle stretching [24] and home exercise programs [23], have shown efficacy in pain control; however, their benefits tend to accrue gradually and depend heavily on patient compliance. This may partly explain why plantar orthotics, although effective, did not achieve the magnitude of improvement observed with neuromodulation.

Emerging therapies such as trigger point therapy combined with ESWT [25], radiofrequency treatments [26], and needle scalpel puncture [27] show promise but currently lack standardized protocols and require further validation. Our study focused on accessible, routinely used treatments in clinical practice, thus supporting their applicability in real-world settings.

Finally, the confirmation of normal data distribution via the Shapiro–Wilk test justified the use of parametric statistics, strengthening the robustness of our findings. The groups were well matched for age, sex, BMI, and symptom duration at baseline (all *p* > 0.60, partial η^2^ < 0.01), suggesting that observed differences in clinical outcomes are attributable to the treatment modalities applied.

**Limitations:** This study’s retrospective design limits causal inference. Potential residual confounding exists despite baseline homogeneity. Absence of subgroup analysis by sex and age may obscure effect modifiers. Findings may not generalize beyond similar clinical settings. Lack of blinding may introduce performance bias.

## 5. Conclusions

Neuromodulation showed greater long-term pain relief at six months compared to shock wave therapy and plantar orthotics, with significant differences and a moderate effect size. All treatments reduced pain significantly at one month, with no short-term differences. Functional recovery was similar across groups. Longer symptom duration and higher BMI were linked to less pain improvement, highlighting the need for early intervention and possibly adjunctive treatments. Neuromodulation is recommended as a first-line option for pain control, while the other therapies remain valid alternatives based on accessibility and patient preference.

## Figures and Tables

**Table 1:** Elegibility Criteria.

Inclusion Criteria	Exclusion Criteria
Age ≥ 18 years.	History of foot/ankle surgery.
Clinical + ultrasonographic diagnosis of plantar fasciopathy.	Coexisting foot musculoskeletal disorders (e.g., neuropathy, fractures).
Received one of the three predefined treatment protocols.	Systemic conditions impairing healing (uncontrolled DM, RA).
Complete pain/function scores (VAS, FFI) at baseline, 1 month, 6 months.	Concurrent therapies not in protocol.
Adherence to institutional treatment pathways.	Incomplete records or loss to follow-up.

**Table 2:** Baseline characteristics of participants by treatment group. Note: ANOVA was used for continuous variables and chi-square test for categorical variable. No significant differences between groups were observed.

	Neuromodulation (n = 35)	Shockwave Therapy (n = 40)	Orthotic Supports (n = 38)	*p* (ANOVA/χ^2^)
Age (years)	46.2 ± 8.1 (95% CI 43.1–49.3)	47.5 ± 7.8 (95% CI 44.8–50.2)	47.8 ± 6.9 (95% CI 44.9–50.7)	0.62 (F(2,110) = 0.48, η^2^ₚ = 0.009)
BMI	28.4 ± 2.3 (95% CI 27.7–29.1)	28.7 ± 2.1 (95% CI 28.1–29.3)	28.5 ± 1.9 (95% CI 27.9–29.1)	0.85 (F(2,110) = 0.16, η^2^ₚ = 0.003)
Symptom duration (months)	14.1 ± 5.2 (95% CI 12.4–15.8)	13.8 ± 4.9 (95% CI 12.3–15.3)	14.3 ± 5.0 (95% CI 12.7–15.9)	0.91 (F(2,110) = 0.10, η^2^ₚ = 0.002)
Sex (% male)	60%	55%	58%	0.89 (χ^2^(2) = 0.22, φ = 0.044)

**Table 3:** Clinical outcomes: pain intensity (VAS), foot function (FFI), and patient satisfaction at each time point. Note: Values are presented as mean ± standard deviation with 95% confidence intervals in parentheses.

	Neuromodulation (Mean ± SD, 95% CI)	Shockwave Therapy (Mean ± SD, 95% CI)	Orthotic Supports (Mean ± SD, 95% CI)
VAS (Pre-treatment)	7.8 ± 1.2 (7.4–8.2)	7.7 ± 1.1 (7.3–8.1)	7.8 ± 1.0 (7.4–8.2)
VAS (1 month)	5.4 ± 1.3 (5.0–5.8)	5.5 ± 1.2 (5.2–5.8)	5.6 ± 1.1 (5.3–5.9)
VAS (6 months)	1.5 ± 1.4 (1.0–2.0)	1.8 ± 1.6 (1.3–2.3)	1.9 ± 1.5 (1.4–2.4)
FFI (Pre-treatment)	76 ± 7 (72–80)	75 ± 6 (72–78)	76 ± 5 (73–79)
FFI (1 month)	52 ± 6 (48–56)	53 ± 5 (50–56)	54 ± 4 (51–57)
FFI (6 months)	30 ± 6 (26–34)	32 ± 5 (29–35)	33 ± 4 (30–36)
Satisfaction	4.2 ± 0.9 (3.8–4.6)	4.0 ± 0.8 (3.7–4.3)	3.9 ± 0.7 (3.6–4.2)

**Table 4:** Within-group clinical evolution (Repeated Measures ANOVA). Note: Partial eta squared values indicate large effect sizes for all groups on both variables.

	Group	*p* (Within-Group)	Partial η^2^ (η^2^ₚ)
VAS	Neuromodulation	<0.001	0.54
	Shockwave Therapy	<0.001	0.49
	Orthotic Supports	<0.001	0.51
FFI	Neuromodulation	<0.001	0.56
	Shockwave Therapy	<0.001	0.52
	Orthotic Supports	<0.001	0.50

**Table 5:** Between-group comparisons at 6 months (one-way ANOVA and Tukey post hoc tests).

	F (df = 2,110)	*p*	Partial η^2^ (η^2^ₚ)	Significant Differences (Tukey Post Hoc)
VAS	4.10	0.02	0.07	Neuromodulation < Shockwave Therapy (*p* = 0.03)
				Neuromodulation < Orthotic Supports (*p* = 0.01)
FFI	2.10	0.12	0.04	Not significant
Satisfaction	1.80	0.17	0.03	Not significant

## Data Availability

Data not available. The datasets generated and analyzed during the current study are not publicly available due to restrictions related to patient confidentiality and data protection regulations. Access to anonymized data may be considered upon reasonable request and with appropriate ethical and institutional approvals.
